# Crystal structure of signal regulatory protein gamma (SIRPγ) in complex with an antibody Fab fragment

**DOI:** 10.1186/1472-6807-13-13

**Published:** 2013-07-04

**Authors:** Joanne E Nettleship, Jingshan Ren, David J Scott, Nahid Rahman, Deborah Hatherley, Yuguang Zhao, David I Stuart, A Neil Barclay, Raymond J Owens

**Affiliations:** 1 Division of Structural Biology, Henry Wellcome Building for Genomic Medicine, University of Oxford, Roosevelt Drive, Oxford OX3 7BN, UK; 2 OPPF-UK, The Research Complex at Harwell, Rutherford Appleton Laboratory, Harwell Oxford, Oxfordshire OX11 0FA, UK; 3 The Research Complex at Harwell and ISIS Neutron and Muon source, Rutherford Appleton Laboratory, Harwell Oxford, Oxfordshire OX11 0FA, UK; 4 The School of Biosciences, University of Nottingham, Sutton Bonington Campus, Sutton Bonington, Leicestershire LE12 5RD, UK; 5 Sir William Dunn School of Pathology, University of Oxford, South Parks Road, Oxford OX1 3RE, UK; 6 Diamond Light Sources, Harwell Science and Innovation Campus, Didcot OX11 0DE, UK

**Keywords:** Antigen-binding complex, Signal regulatory protein, Receptor structure

## Abstract

**Background:**

Signal Regulatory Protein γ (SIRPγ) is a member of a closely related family of three cell surface receptors implicated in modulating immune/inflammatory responses. SIRPγ is expressed on T lymphocytes where it appears to be involved in the integrin-independent adhesion of lymphocytes to antigen-presenting cells. Here we describe the first full length structure of the extracellular region of human SIRPγ.

**Results:**

We obtained crystals of SIRPγ by making a complex of the protein with the Fab fragment of the anti-SIRP antibody, OX117, which also binds to SIRPα and SIRPβ. We show that the epitope for FabOX117 is formed at the interface of the first and second domains of SIRPγ and comprises residues which are conserved between all three SIRPs. The FabOX117 binding site is distinct from the region in domain 1 which interacts with CD47, the physiological ligand for both SIRPγ and SIRPα but not SIRPβ. Comparison of the three domain structures of SIRPγ and SIRPα showed that these receptors can adopt different overall conformations due to the flexibility of the linker between the first two domains. SIRPγ in complex with FabOX117 forms a dimer in the crystal. Binding to the Fab fixes the position of domain 1 relative to domains 2/3 exposing a surface which favours formation of a homotypic dimer. However, the interaction appears to be relatively weak since only monomers of SIRPγ were observed in sedimentation velocity analytical ultracentrifugation of the protein alone. Studies of complex formation by equilibrium ultracentrifugation showed that only a 1:1 complex of SIRPγ: FabOX117 was formed with a dissociation constant in the low micromolar range (*K*_d_ = 1.2 +/− 0.3 μM).

**Conclusion:**

The three-domain extracellular regions of SIRPs are structurally conserved but show conformational flexibility in the disposition of the amino terminal ligand-binding Ig domain relative to the two membrane proximal Ig domains. Binding of a cross-reactive anti-SIRP Fab fragment to SIRPγ stabilises a conformation that favours SIRP dimer formation in the crystal structure, though this interaction does not appear sufficiently stable to be observed in solution.

## Background

Members of the signal regulatory protein family (SIRP) play important roles in the regulation of the immune response in man [1]. The family comprises three type I transmembrane glycoproteins (α, β, γ) each with an extracellular region consisting of three Ig-like domains followed by a single transmembrane sequence and a cytoplasmic domain which varies in length between the three SIRPs. SIRPs are classified as “paired receptors” since they show the following characteristics: (1) they are encoded by different genes arranged in a gene cluster, (2) they share significant sequence homology in their extracellular domains and (3) they comprise both activating and inhibitory members. Thus SIRPα delivers an inhibitory signal via immunoreceptor tyrosine-based inhibition motifs (ITIMs) located in the cytoplasmic domain of the protein as well as interacting with the ligand CD47 [2]. SIRPβ delivers an activating signal through association with DAP12, a transmembrane adaptor protein with an immunoreceptor tyrosine-based activation motif (ITAM), but does not bind to CD47 [1]. By contrast, SIRPγ appears to have no signaling function but does bind to CD47, though this interaction is ten times weaker than that of SIRPα [3]. Unlike the other SIRP proteins, SIRPγ is expressed by T cells where it interacts with CD47 on the surface of the cell, resulting in increased cell-cell adhesion in an integrin-independent manner [4]. Therefore, it is thought that SIRPγ may be involved in T cell responses as an accessory protein [4]. Note, SIRPγ was originally called SIRPβ2 but this term is no longer used [5].

There has been considerable interest in the structure of the SIRP family with regard to the subtle differences in ligand binding specificity [6,7] and a putative relationship to primitive antigen receptors [8]. Crystal structures have been determined for the N terminal domains of SIRPα [9,10], SIRPβ and SIRPγ [11], a full extracellular region of SIRPα [12] and a complex of the N-terminal domain of SIRPα and CD47 [11].

In this paper we present the X-ray crystal structure of the complete extracellular portion of SIRPγ co-crystallized with the Fab fragment of the OX117 monoclonal antibody, which recognises SIRPα, SIRPβ and SIRPγ [3]. The Fab fragments of antibodies have been used extensively as co-crystallization chaperones [13,14] and binding to Fab fragments of OX117 facilitated the crystallization of SIRPγ in this study. The eptiope recognised by OX117 involved residues in both domains 1 and 2 (d1 and d2) of SIRPγ and was distinct from the CD47 binding site on d1. Interestingly, SIRPγ formed a dimer in the crystal structure through an interface between d1 and d2.

## Results and discussion

### Overall structure

**Figure 1:**
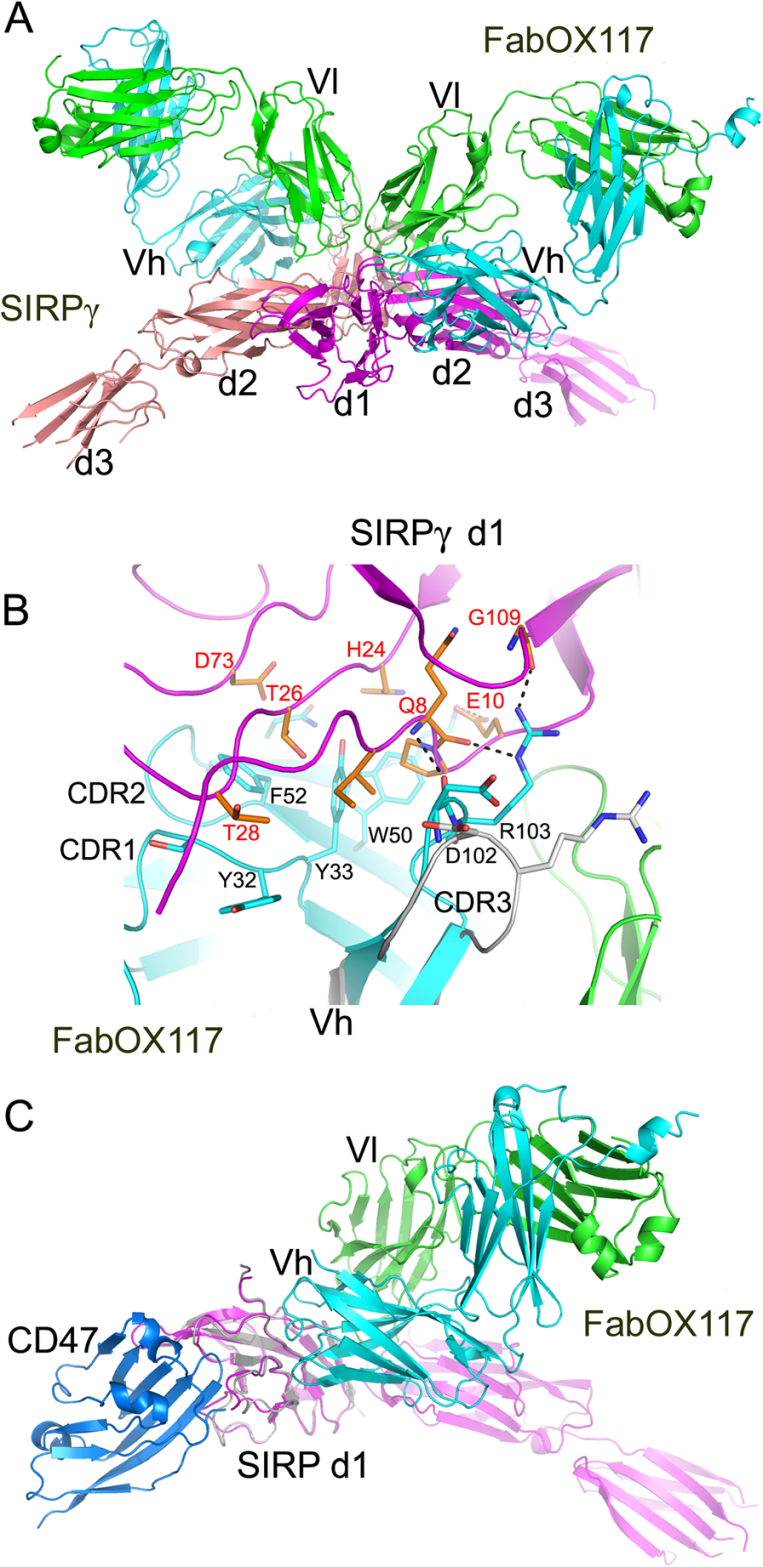
**Structure of the FabOX117: SIRPγ complex. (A)** Overview of the FabOX117: SIRPγ complex showing two SIRPγ (coloured magenta and pink) molecules forming a dimer in the asymmetric unit each bound to a Fab fragment of the monoclonal antibody, OX117 [3], (heavy chain coloured cyan, light chain coloured green) **(B)** Overlay of bound/unbound (PDB id **3DIF**^15^) FabOX117 showing the change in position of CDR3 of the heavy chain variable domain in bound (cyan) compared to the unbound (grey) FabOX117. The key residues involved in the interaction between Vh and d1 of SIRPγ (coloured orange) are shown in stick representation. **(C)** Comparison of SIRPγ: FabOX117 (coloured as Figure 1A) and SIRPα: CD47 complexes (coloured grey and blue respectively) showing that both CD47 and FabOX117 can bind to SIRPγ d1 at the same time.

In contrast to SIRPα [12], attempts to grow diffraction quality crystals of the full three domain extracellular region of SIRPγ proved unsuccessful. Therefore an antibody chaperone approach was adopted using the Fab fragment of a SIRP monoclonal antibody (OX117) that had previously been generated [3,15]. Both Fab fragments of OX117 and the extracellular region of SIRPγ were produced as recombinant proteins in mammalian cells. Diffracting crystals of the FabOX117: SIRPγ complex were successfully obtained following de-glycosylation of the SIRPγ: FabOX117 protein complex using endoglycosidase F1. The structure of the complex was solved to 2.5 Å resolution by molecular replacement. The crystallographic asymmetric unit contains a SIRPγ dimer, with each SIRPγ monomer bound to a FabOX117 (Figure 1A). The final refined model has a R-factor of 0.200 (R-free: 0.259) with root mean square deviations (rmsds) of 0.007 Å for bond lengths and 1.2° for bond angles from the ideal values. The C-terminal end of d3 of SIRPγ is flexible, residues 294–295 and 317–332 in one molecule, and residues 232–239, 289–298 and 316–332 in another are not defined in the electron density map and not included in the model. The two SIRPγ molecules can be superimposed with a rmsd of 0.61 Å for 294 equivalent Cα atoms, while the rmsd is 0.63 Å for 428 equivalent Cα atoms between the two FabOX117 molecules. Of the 4 potential glycosylation sites in each SIRPγ molecule, one at residue 240 has sufficient density allowing the N-linked N-acetylglucosamine to be modelled; weak electron density attached to residue 215 suggests the presence of glycosylation. There is no evidence in the electron density map for the modification at Asn281 and Asn289, which contrasts with the observations reported for the structure of SIRPα [12]. The C-terminal His_6_ tag from one of the Fab heavy chains is ordered, presumably due to crystal contacts. Two His residues, at position 1 and 5, together with a water molecule and His56 from a symmetry related SIRPγ bind a zinc ion with the canonical tetrahedral coordination.

### The Fab binding site

From the structure it can be seen that the antibody Fab fragment binds to both d1 and d2 of SIRPγ (Figure 1A). The light chain variable region (Vl) appears to be inserted between the two domains contacting residues in both d1 and d2, whereas the heavy chain variable domain (Vh) only interacts with d1. Comparison of bound and unbound FabOX117 (PDB id 3DIF) shows only small changes in conformation upon binding (Figure 1B). Alignment using PDBeFold [16] of the Fd region (Vh and CH1 domains) gives a rmsd of 0.6 Å for the Cα backbone for 208 out of 220 residues. Regions of the Fd fragment that were disordered in the unbound Fab structure, namely the Vh loop consisting of residues G133 to S140 and the C-terminal residues 219 to 220 become ordered on binding to the antigen. For the light chain the rmsd for the Cα backbone is 0.7 Å for 213 out of 214 residues. The most significant conformational change in FabOX117 upon binding to SIRPγ is the re-positioning of the side-chain of R103 in the third Complementarity Determining Region (CDR3) of Vh (Figure 1B). R103 forms a network of hydrogen bonds with SIRPγ d1 involving Q8 and E10 in the loop connecting β-strands A1 and A2 and G109 in the G1/G2 loop (Figure 1B). Additional hydrogen bond interactions between FabOX117 and SIRPγ are observed between Y92 in Vl CDR3 and K11 and L12 in the A1/A2 loop of SIRPγ and between S26 of Vl CDR1 and D149 in d2 of SIRPγ. All these residues are conserved between SIRPα, β and γ indicating that OX117 would bind in a similar way to all three SIRPs, consistent with the cross-reactivity data previously published for this antibody [3].

The structure of the isolated d1 domain of SIRPγ has been solved previously (PDB id 2JJW [11]). Overlaying this structure onto the full length SIRPγ, showed that binding of the Fab fragment to d1 did not change the conformation of the domain. Thus alignment of the two d1 structures gave a rmsd for the Cα backbone of 0.5 Å for 109/116 amino acids, with the loop comprising G97 to E100 becoming ordered in the complex. Overall, binding of the FabOX117 to SIRPγ appears to result in very little conformational change in either the antibody or antigen.

The crystal structure of d1 of SIRPα in complex with CD47 has been solved by Hatherley *et al*. [11]. When the d1 of the FabOX117: SIRPγ crystal structure is aligned with the SIRPα(d1): CD47 structure (PDB id 2JJS), the locations where Fab and CD47 bind to SIRPγ do not overlap (Figure 1C). The main contact residues for CD47 are in three loops connecting strands B2-C, D-E and F-G1. This suggests that SIRPγ could bind both FabOX117 and CD47 at the same time.

### Comparison of SIRPγ with SIRPα

**Figure 2:**
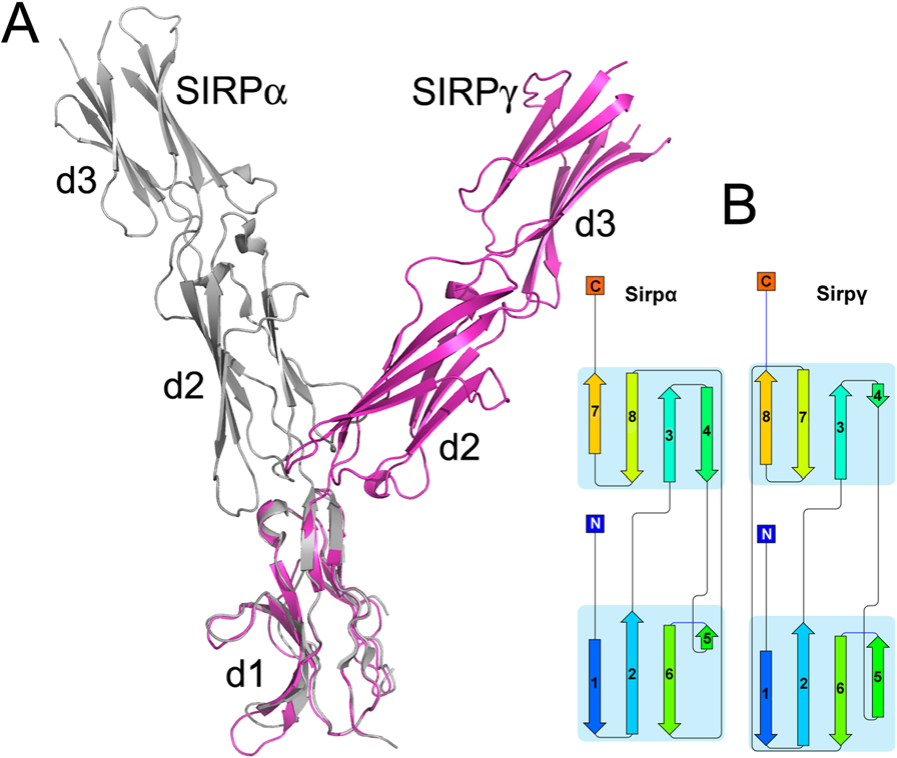
**Comparison of the structure of SIRPα and SIRPγ. (A)** Overlay of SIRPα (PDB id 2WNG [12]) (coloured grey) SIRPγ (coloured magenta) and **(B)** Cartoon of the secondary structure of the d3 domains of SIRPα and SIRPγ.

Comparison of the full length extracellular three domain structure of SIRPγ with that of SIRPα (PDB id 2WNG [12]) shows a high level of structural similarity for the individual domains (d1-d3) (Figure 2A). One small difference is the relative length of the C’ and D strands in the d3 domains which are short and long respectively in SIRPγ and the opposite in SIRPα, though this does not alter the overall topology of the d3 domains (Figure 2B). However, there is a significant difference in the relative orientation of the three domains between the two SIRP structures (Figure 2A). Thus, in the superimposition of d1 of SIRPγ onto the d1 of SIRPα (rmsd of 0.60 Å for 105 Cα atoms out of 114 residues), the d2-d3 domains of the two proteins are rotated by 66° with respect to each other (Figure 2A). The difference in the relative arrangement of d1 and d2-d3 in the two SIRPs would affect the ability of SIRPα to bind to FabOX117 since the interactions between FabOX117 VL and d2 observed in SIRPγ would not be possible in the SIRPα structure shown in Figure 2 (PDB id 2WNG). This suggests that there is some flexibility in the d1-d2 inter-domain region as OX117 has previously been shown to bind to all three SIRPs [3].

**Figure 3:**
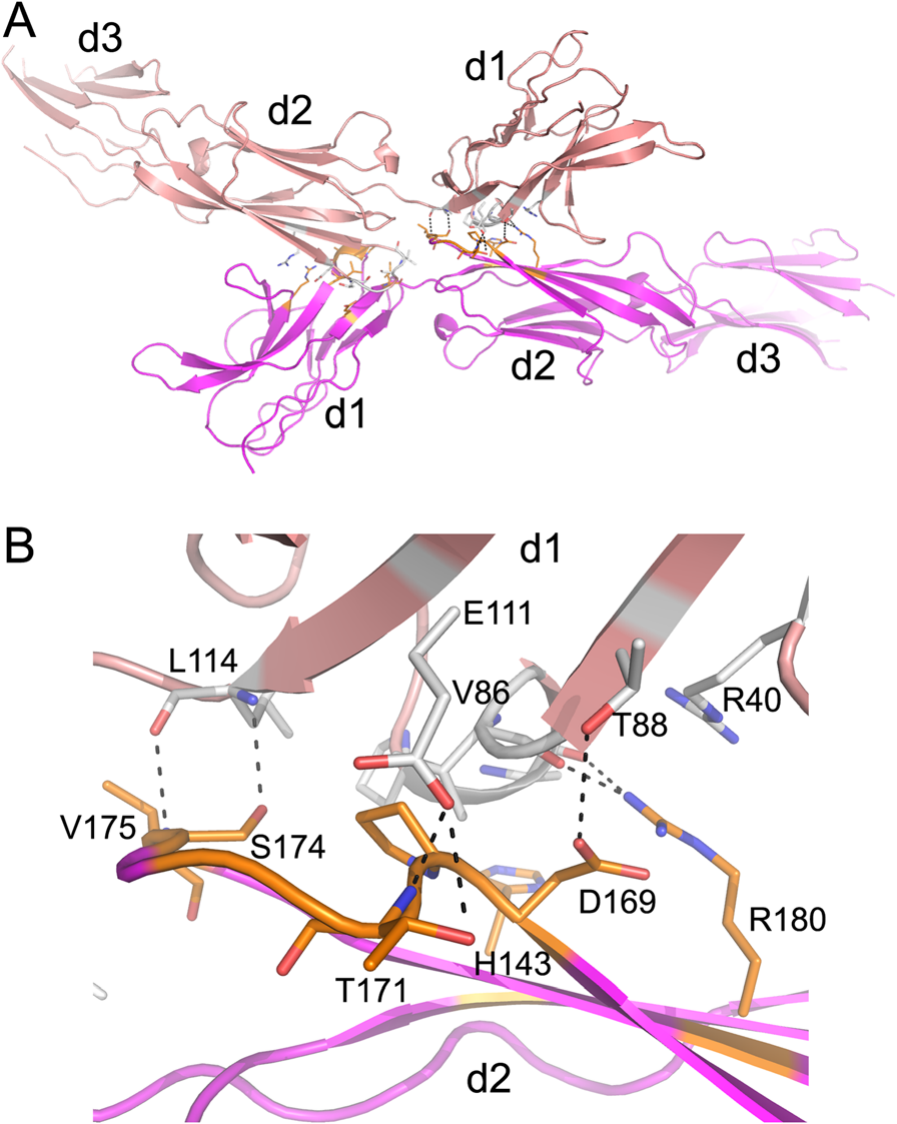
**SIRPγ dimer interface. (A)** SIRPγ dimer showing the head-to-head interaction between the adjacent SIRPγ molecules (coloured pink and magenta) **(B)** Close-up of the SIRPγ dimer interface between domains 1 (d1) and 2 (d2) with residues that contribute to hydrogen-bonding shown in stick representation (d1 coloured grey and d2 coloured orange).

Interestingly, in contrast to the structure of SIRPα alone [12], the two SIRPγ molecules bound to FabOX117 formed a head-to head dimer in the crystal structure (Figure 1A) with a contact area of about 1130 Å^2^ and a Complex Formation Significance Score [17] of 0.78. This is at the lower end of the range considered to be physiological significant (1600 +/− 400 Å^2^) [18] though it is comparable to the contact surface area (1150 Å^2^) and significance score (0.74) for the SIRPγ: Fab interface. It therefore appears to be non-trivial. The two SIRPγ molecules form an arch-like structure spanning a distance of approximately 160 Å with the Fab fragments binding to the outer surface of the arch (Figure 3A). Dimer formation appears to be a consequence of the relative orientation of d1 with respect to d2 which is imposed by the Fab binding. The interface between the two SIRPγ chains comprises a number of hydrogen-bond interactions between residues in d1 and d2, e.g. T88-D169, L114-S174, E111-G172 and a salt bridge R180-E47 (Figure 3B). Six of these interface residues are conserved between all three SIRP sequences, with the other two showing relatively conservative substitutions (R180H and L114V).

### Analysis of the SIRPγ: FabOX117 complex by analytical ultracentrifugation

**Figure 4:**
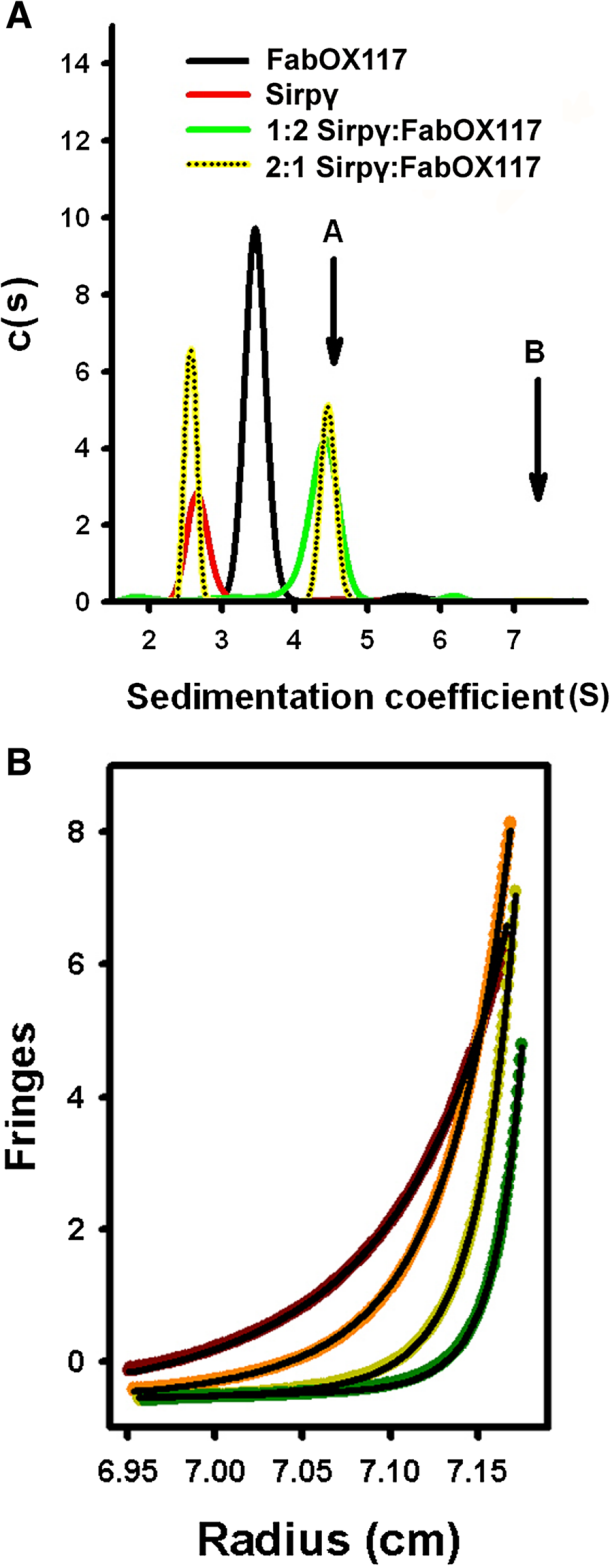
**Analytical ultracentrifugation of SIRPγ, FabOX117 and SIRPγ: FabOX117 complexes. (A)** Sedimentation velocity distributions for SIRPγ, FabOX117, a 2:1 and a 1:2 mixture of SIRPγ: FabOX117. Initial sedimentation distributions were analysed in SEDFIT, whereas data were subsequently fitted in SEDANAL [22]. **(B)** Sedimentation equilibrium data of SIRPγ: FabOX117 were obtained at 2:1, 1:1 and 1:2 stoichiometric ratios; for clarity, only the data at 2:1 ratio is shown. Sedimentation equilibrium was attained at 15 000 rpm (black), 20 000 rpm (red), 28 000 rpm (green) and 36 000 rpm (yellow with black dots). Data were extracted with SEDFIT [22] and analysed in SEDPHAT [23]. A single species model did not fit the data. The data were then fitted to an A + B ↔ C model where A is SIRPγ, B is FabOX117 and C is the 1:1 complex; this fit is represented by the solid black line going through each of the data traces.

The observation that SIRPγ is dimeric in the crystal, poses the question as to whether this self-association occurs in solution. Sedimentation velocity analytical ultracentrifugation experiments were carried out on both SIRPγ and FabOX117 proteins individually. Experimentally determined sedimentation coefficients, frictional ratios and molecular weights were compared to values calculated from the coordinates of their crystal structures using US-SOMO [19] (Table 1). The results showed that SIRPγ was monodisperse and behaved as a monomer. A single species was also observed for FabOX117 which corresponded to the assembled light and heavy chain. Sedimentation velocity experiments of a 2:1 stoichiometric mixture of SIRPγ: FabOX117 gave two peaks at 2.70 *S* and 4.85 *S* (Figure 4A). The first peak was consistent with the calculated and measured value for SIRPγ alone, whilst the second peak corresponded to the sedimentation coefficient calculated for a 1:1 complex of SIRPγ and FabOX117 (Table 1; marked ‘A’ in Figure 4A). A 1:2 mixture of SIRPγ and FabOX117 gave a single skewed distribution around a sedimentation coefficient of 4.5 *S* (Figure 4A), close to that determined for the 1:1 complex. We interpret this result as follows: under the conditions where there is a two-fold molar excess of the FabOX117 over the SIRPγ protein, a 1:1 complex with SIRPγ is formed, which partially dissociates during sedimentation. Since the free FabOX117 has a similar sedimentation coefficient to the FabOX117: SIRPγ complex (4.85 *S* compared to 4.5 *S*) there is no resolution of the bound and unbound FabOX117 into individual peaks, and a skewed composite peak observed. However the sedimentation coefficient of SIRPγ alone is sufficiently different from the complex (2.5 *S* vs. 4.5 *S*) that a peak corresponding to the SIRPγ alone can be resolved (Figure 4A). The theoretical basis for this behaviour has been described previously by Cann, Winzor and co-workers [20,21]). Nonetheless, if the SIRPγ: FabOX117 formed a 2:2 complex, as observed in the crystal structure, then a species sedimenting at 7.25 *S* would be expected (see Table 1; marked ‘B’ in Figure 4A). However no such higher order species were observed in any of the sedimentation velocity experiments.

**Table 1:** Hydrodynamic parameters

***Sample***	$M_{\mathrm{app}}^{0}$ ^***a***^	$S_{20,w}^{0}$ ^***b***^	${\left(\frac{f}{f_{0}}\right)}_{\mathrm{app}}$ ^***b***^	***M***_***calc***_^***c***^	***S***_***calc***_^***c***^	${\left(\frac{f}{f_{0}}\right)}_{\mathrm{calc}}$ ^***c***^
Fab	51.2	3.7	1.4	48.4	3.81	1.22
Sirp	39.4	2.7	1.6	42.6	2.52	1.46
1:1 Complex	91.0^d^	4.8	1.5	91.0	4.85	1.42
2:2 Complex	Nd	Nd	Nd	182.0	7.25	1.48

^a^calculated using SEDANAL. Units are in kilodaltons.

^b^determined from sedimentation velocity. Units are in S.

^c^calculated from the PDB file using US-SOMO. Units are the same for the observed parameters.

*Nd* not determined.

To assess the strength of the SIRPγ: FabOX117 interaction, sedimentation equilibrium centrifugation experiments were carried out on 2:1, 1:1 and 1:2 stoichiometric mixtures of SIRPγ: FabOX117. Data were extracted using SEDFIT v14.1 [22] and processed with SEDPHAT [23]. The results did not fit a single species model indicating that multiple forms were present in solution (Figure 4B). However, the data fitted well to an A + B ↔ AB model. From this model the derived dissociation constant was determined to be 1.2 (± 0.3) μM, indicating only a moderately strong antibody: antigen interaction. Such a value would also explain the behaviour of the sedimentation velocity profiles seen in Figure 4A, since at the loading concentration used, there would be a small amount of dissociation into individual components giving rise to skewed peaks in the c(s) plot (Figure 4A).

We conclude that the 2:2 SIRP: FabOX117 complex observed in the crystal structure is not formed in solution under the conditions of the AUC experiments. It is important to note that the highest protein concentration of the SIRPγ: FabOX117 mixture used in the AUC experiments was 1 mg/ml (corresponding to 13.7 μM) compared to 16.8 mg/ml (230 μM) for crystallization. Therefore it is possible that the 2:2 complex predominates at high concentrations of the SIRPγ FabOX117 mixture. However we calculate that this would require that the dissociation constant of the 2:2 complex is </= 10 μM. If this was the case then it is surprising that no 2:2 complex was observed at the concentration of 13.7 μM used in the sedimentation velocity experiments. The results of the AUC analyses imply that self-association of SIRP γ only occurs, if at all, at very high protein concentrations and therefore is unlikely to be physiologically relevant.

## Conclusions

A cross-reactive anti-SIRP monoclonal antibody (OX117) has proved useful in crystallizing human SIRPγ and subsequently obtaining the structure of the antibody: antigen complex. This has enabled the cross-reactive epitope to be mapped to the first and second domains of SIRPγ distal from the CD47 ligand binding site on domain 1. The structure of the FabOX117: SIRPγ complex has also revealed the potential for SIRPs to form head-to-head dimers through an interaction between the domains 1 and 2 on adjacent molecules in the crystal. However this interaction was not observed in solution, so its physiological significance is questionable. Comparison of the three domain structures of SIRPγ and SIRPα showed that these receptors can adopt different overall conformations due to the flexibility of the linker between the first two domains. Given the sequence similarity between all members of the SIRP family it seems likely that this is a property shared by all of the receptors.

## Methods

### Protein production

SIRPγ and the Fab fragment of the anti-SIRP monoclonal antibody, OX117 [3] (FabOX117), were expressed as recombinant proteins in mammalian cells. FabOX117 was produced by co-transfection of HEK 293 T cells (available from the American Type Culture Collection as CRL-11268) with vectors encoding the heavy and light chain genes using a CompacT SelecT robotic system (The Automation Partnership, Royston, UK) [24]. The Fab fragment was purified from the cell culture media by nickel affinity chromatography followed by size exclusion chromatography [15]. The extracellular region of SIRPγ was expressed using the SIRPα leader sequence residues 1–30 (accession number CAA71403) followed by residues 29–347 of SIRPγ (accession number NP_061026) with a C-terminal tag of STRHHHHHH using the pEE14 vector in CHO Lec3.2.8.1 cells [25] as previously described [12]. N-glycosylation in these cells is arrested at the high mannose state enabling de-glycosylation by treatment with endoglycosidase H/F1. The SIRPγ was purified from the cell culture media using nickel affinity chromatography followed by size exclusion chromatography [26]. The FabOX117 and SIRPγ proteins were mixed in a 1:1 molar ratio and incubated overnight at 4°C. The FabOX117: SIRPγ complex was treated with Endo F1 to remove the N-linked glycans then purified by size exclusion chromatography.

### Crystallization and structure solution

The FabOX117: SIRPγ (16.8 mg/ml) complex was crystallized from 0.2 M magnesium acetate tetrahydrate, 20% w/v polyethylene glycol 3350 (Hampton PEG/Ion Screen #25). Data were collected to 2.5 Å resolution at Diamond Light Source beamline I03 from a single crystal. Diffraction images each of 1.0° oscillation and 0.5 s exposure were recorded on a ADSC Quantum 315 CCD detector at a X-ray wavelength of 0.9763 Å. The crystal was soaked in a cryoprotectant solution containing 20% (v/v) glycerol and 80% (v/v) crystallization reservoir solution for about 10 s before being plunged into liquid nitrogen and maintained at 100 K under a stream of nitrogen gas during data collection. Data were indexed, integrated and scaled using HKL2000/SCALEPACK [27]. The crystal belongs to a space group of *P*2_1_2_1_2 with unit cell dimensions of *a* = 140.4 Å, *b* = 174.2 Å and *c* = 81.7 Å. The solvent content is 54% by assuming 2 complexes in one crystallographic asymmetric unit.

The structure of the FabOX117: SIRPγ complex was solved by molecular replacement using program MOLREP [28] and search models FabOX117 and SIRPα (PDB IDs: 3DIF and 2WNG). The orientations and positions of Fabs were readily determined, while the domains of SIRPγ had to be found separately by using individual domains of SIRPα as search models. Structure refinement and model rebuilding were carried out with REFMAC [29] and COOT [30] respectively. Tight backbone and loose side-chain NCS restraints were applied throughout the refinement. Diffraction data and structure refinement statistics are shown in Table 2. Structural comparisons used SHP [31]. Structural images for figures were prepared with PyMOL (http://www.pymol.org/). The coordinates and structure factors have been deposited in the Protein Data Bank under accession number 4I2X.

**Table 2:** **X**-**ray data collection and refinement statistics**

***Data collection***	
X-ray source	Diamond I03
Wavelength (Å)	0.97630
Space group	*P*2_1_2_1_2
Unit cell (Å)	*a* = 140.4, *b* = 174.2, *c* = 81.7
Resolution range (Å)	30.0 – 2.50 (2.59-2.50)
Unique reflections	71197 (7000)
Completeness (%)	100 (100)
Redundancy	12.3 (10.5)
Average *I*/*σI*	14.9 (2.1)
R_merge_	0.157 (−−)
*Refinement*	
Resolution range (Å)	30.0 – 2.50
No. of atoms (protein/other atoms)	11212/323
Rms bond length deviation (Å)	0.008
Rms bond angle deviation (°)	1.2
Mean B-factor (protein/other atoms[Å^2^])	38/54
Residues in preferred regions (%)	1112 (89.5)
Residues in allowed regions (%)	128 (10.3)
Residues in disallowed regions (%)	3 (0.2)

^a^R_work_ and R_free_ are defined by R = *Σ*_*hkl*_||*F*_*obs*_| − |*F*_*calc*_||/*Σ*_hkl_|F_obs_|, where *h*,*k*,*l* are the indices of the reflections (used in refinement for R_work_; 5%, not used in refinement, for R_free_), F_obs_ and F_calc_ are the structure factors, deduced from measured intensities and calculated from the model, respectively.

### Analytical ultracentrifugation

Analytical ultracentrifuge experiments were performed on a Beckman-Coulter Proteome Lab XL-I running version 5.8 of the data collection software. Data was obtained using both absorbance and interference optics. Sedimentation velocity data was obtained at 40 000 rpm in 2 channel meniscus-matching centrepiece cells (SpinAnalytical, NH, USA), while sedimentation equilibrium data was obtained at 15 000, 20 000, 28 000 and 36 000 rpm in 2 channel centrepieces (Beckman, USA).

Data were scanned every 2 hours until equilibrium had been reached, as determined by calculated RMSDs between successive scans using SEDFIT v14.1 and analysed using SEDPHAT. Loading concentrations were 1.0, 0.2, 0.1 mg/ml for Sirpγ and FabOX117 alone. These corresponded to molar loading concentrations of 20, 4 and 2 μM for Sirpγ and 23, 4.2 and 2.1 μM for FabOX117. For the mixtures, these were performed at a total concentration of 1 mg/ml at molar ratios of 1:2, 1:1 and 2:1, respectively. These corresponded to molar loading concentrations of 6.3/13.7 μM, 10/10 μM and 13.7/6.3 μM. The buffer used throughout was 20 mM Tris–HCl pH 7.5 200 mM NaCl. The solvent density and viscosity were calculated to be 1.0070 and 1.002, respectively using SEDNTERP [32]. The molecular weight and partial specific volumes for FabOX117 was calculated using SEDNTERP to be 48 380 Da and 0.7281 g/ml, respectively. The corresponding values for SIRPγ were calculated on the basis of an additional 1.8 kDa of glycosylation to the protein sequence and found to be 52 800 Da and 0.7321 g/ml, respectively. All data were obtained at 20°C.

### Data analysis of analytical ultracentrifuge data

Sedimentation velocity data were analysed using SEDFIT v14.1, and sedimentation coefficients determined from the weight averaged integration of the peaks using the integration functions contained in the software. Corrected sedimentation coefficients (S_20, w_ values) were noted for each loading concentration and used to extrapolate back to infinite dilution to obtain values for $S_{20.w}^{0}$ for each species. Sedimentation equilibrium data were excised using SEDFIT v14.1 and then exported into SEDPHAT. Data were then fitted to a heterogeneous association A + B ⇔ AB, where A = FabOX117 (light and heavy chain) and B = SIRPγ monomer. Errors in the determined dissociation constant were calculated within SEDPHAT using a Monte Carlo routine, and errors were quoted at the 95% confidence level.

## Competing interests

The authors declare that they have no competing interest.

## Authors’ contributions

ANB and RJO initiated the study. JN, DH, NR, YZ produced the proteins, JN crystallized the complex, JR collected and processed the diffraction data, modeled and refined the structure. JR, ANB, and RJO analyzed the structure. DJS carried out AUC measurements and analyzed the data. JR, ANB, DIS, RJO and DJS wrote the paper. All authors read and approved the final manuscript.
